# The Wheat Wall-Associated Receptor-Like Kinase TaWAK-6D Mediates Broad Resistance to Two Fungal Pathogens *Fusarium pseudograminearum* and *Rhizoctonia cerealis*

**DOI:** 10.3389/fpls.2021.758196

**Published:** 2021-10-27

**Authors:** Haijun Qi, Feilong Guo, Liangjie Lv, Xiuliang Zhu, Li Zhang, Jinfeng Yu, Xuening Wei, Zengyan Zhang

**Affiliations:** ^1^The National Key Facility for Crop Gene Resources and Genetic Improvement, Institute of Crop Sciences, Chinese Academy of Agricultural Sciences, Beijing, China; ^2^Institute of Cereal and Oil Crops, Hebei Academy of Agriculture and Forestry Sciences, Shijiazhuang, China; ^3^College of Plant Protection, Shandong Agricultural University, Taian, China

**Keywords:** broad resistance, common wheat (*Triticum aestivum*), *Fusarium pseudograminearum*, *Rhizoctonia cerealis*, wall-associated receptor-like kinase

## Abstract

The soil-borne fungi *Fusarium pseudograminearum* and *Rhizoctonia cerealis* are the major pathogens for the economically important diseases *Fusarium* crown rot (FCR) and sharp eyespot of common wheat (*Triticum aestivum*), respectively. However, there has been no report on the broad resistance of wheat genes against both *F. pseudograminearum* and *R. cerealis*. In the current study, we identified *TaWAK-6D*, a wall-associated kinase (WAK) which is an encoding gene located on chromosome 6D, and demonstrated its broad resistance role in the wheat responses to both *F. pseudograminearum* and *R. cerealis* infection. *TaWAK-6D* transcript induction by *F. pseudograminearum* and *R. cerealis* was related to the resistance degree of wheat and the gene expression was significantly induced by exogenous pectin treatment. Silencing of *TaWAK-6D* compromised wheat resistance to *F. pseudograminearum* and *R. cerealis*, and repressed the expression of a serial of wheat defense-related genes. Ectopic expression of *TaWAK-6D* in *Nicotiana benthamiana* positively modulated the expression of several defense-related genes. TaWAK-6D protein was determined to localize to the plasma membrane in wheat and *N. benthamiana*. Collectively, the TaWAK-6D at the plasma membrane mediated the broad resistance responses to both *F. pseudograminearum* and *R. cerealis* in wheat at the seedling stage. This study, therefore, concludes that *TaWAK-6D* is a promising gene for improving wheat broad resistance to FCR and sharp eyespot.

## Introduction

Wheat is one of the most important staple crops as it provides about a fifth of the total calories consumed by humans and contributes more protein than any other food source (Consortium, 2018). Fungal diseases comprise one of the most serious threats to wheat (*Triticum aestivum*) production. The sharp eyespot disease, caused by the necrotrophic fungus *Rhizoctonia cerealis*, and *Fusarium* crown rot (FCR), caused mainly by the soil-borne fungus *Fuusarium pseudograminearum*, are destructive diseases of wheat in many regions of the world (Hamada et al., 2011; Li et al., 2012; Chen et al., 2013; Kazan and Gardiner, 2018). Planting resistant wheat cultivars is the most environmentally friendly and effective method to control both two diseases. Yet, no wheat germplasm with complete resistance has been found until now. Only a few quantitative trait loci (QTL) were reported to confer resistance to sharp eye spots or FCR in wheat (Smiley and Yan, 2009; Chen et al., 2013; Wu et al., 2017; Yang X. et al., 2019). For example, one of the most important FCR-resistant QTL is located on the long arm of chromosome 3B (3BL) in a recombinant inbred line (RIL) population of “CSCR6/Lang” and explained the phenotypic variation up to 49% in Australia (Ma et al., 2010). Genome-wide association studies (GWAS) on 234 Chinese wheat cultivars revealed that 286 SNPs to be significantly associated with FCR resistance, of which 266, 6, and 8 were distributed on chromosomes 6A, 6B, and 6D FCR-resistant QTLs, respectively (Yang X. et al., 2019). Additional studies have identified significant QTLs on various chromosomes, e.g., 2B, 3B, 4B, 4D, and 7A in RIL populations “Sunco/Macon” and “Sunco/Otis” (Poole et al., 2012). A recent study reported that by virus-inducing gene silencing (VIGS) and ethyl methanesulfonate (EMS) mutant, the loss-of-function of the wheat dirigent gene *TaDIR-B1* increases host resistance to FCR (Yang et al., 2021). However, works of research about the functional role of FCR resistance-related genes are still rare.

Plants have evolved two layers of innate immune systems to defend against microbial pathogens, including pathogen-associated molecular pattern (PAMP) triggered immunity and effector-triggered immunity (Jones and Dangl, 2006). Plants deploy a large number of receptor-like kinases (RLKs) and receptor-like proteins (RLPs) as pattern recognition receptors (PRRs) that detect microbe- and host-derived molecular patterns as the first layer of inducible defense (Zipfel, 2014; Couto and Zipfel, 2016; Tang et al., 2017; Rhodes et al., 2021). Wall-associated kinases (WAKs) are a unique group of RLKs, characterized by an aminol (N)-terminal signal peptide, an extracellular WAK galacturonan-binding (GUB) domain, an extracellular epidermal growth factor (EGF)-like domain, an EGF calcium-binding (EGF-Ca^2+^) domain, a transmembrane (TM) domain, and a carboxyl (C)-terminal cytosolic Ser/Thr protein kinase domain (Verica and He, 2002). WAKs were shown to likely link to the cell wall through the homogalacturonan binding domain (Kanneganti and Gupta, 2008; Delteil et al., 2016). In *Arabidopsis, AtWAK1* and *AtWAK2* were shown to bind to pectin molecules resident in native cell walls (Decreux and Messiaen, 2005; Kohorn et al., 2009). *AtWAK1* might act as a receptor of oligogalacturonides (OGs), released from the plant cell wall during pathogen infection (Brutus et al., 2010).

Several groups reveal that *WAKs* are important in resistance responses to bacterial and fungal diseases. In *Arabidopsis thaliana, AtWAK1* is required for the resistance to *Botrytis cinerea* (Brutus et al., 2010), and *WAKL22* confers the dominant resistance to *Fusarium* wilt disease (Diener and Ausubel, 2005). In rice, the recently-cloned disease resistance gene *Xa4*, which encodes a WAK, confers durable resistance against *Xanthomonas oryzae* pv. *oryzae* and promotes cellulose synthesis, suppresses cell wall loosening, and strengthens the plant cell wall (Hu et al., 2017). Besides, the *OsWAK1, OsWAK14, OsWAK91*, and *OsWAK92* were shown to positively participate in resistance to the blast fungus *Magnaporthe oryzae* (Li et al., 2009; Cayrol et al., 2016). Overexpression of *OsWAK25* enhanced resistance to both the hemibiotrophic pathogens *X. oryzae* and *M. oryzae* in rice (Harkenrider et al., 2016). Two distinct WAK genes from maize (*Zea mays*), namely *Htn1* and *ZmqHSR1*, were map-cloned and verified as important quantitative resistance loci for resistance against the foliar fungal pathogen *Exserohilum turcicum* and the soil-borne fungus *Sporisorium reilianum* (Hurni et al., 2015; Zuo et al., 2015; Yang et al., 2019). The most recent research revealed that the cotton WAK *GhWAK7A* mediates resistance responses to fungal wilt pathogens by complexing with the chitin sensory receptors *GhLYK5* and *GhCERK1* (Wang P. et al., 2020). In wheat, the disease resistance gene *Stb6*, which locates on the short arm of chromosome 3A and encodes a WAK-like protein, confers resistance to *Septoria tritici* blotch (STB) disease caused by *Zymoseptoria tritici* (Saintenac et al., 2018). Additionally, the WAK-encoding gene *TaWAK6*, located on wheat chromosome 5B, confers adult plant resistance to leaf rust (*Puccinia triticina*) (Dmochowska-Boguta et al., 2020). However, the function of *WAK* genes involved in broad resistance to fungal pathogens in wheat was scarcely reported.

In this current research, we mined a wheat *WAK* gene *TaWAK-6D* in the wheat response to both fungal pathogens and investigated the predicted subcellular localization of the protein and the functional role in wheat resistance responses to infection of *R. cerealis* and *F. pseudograminearum*. These results suggested that TaWAK-6D, distributed at the plasma membrane, could modulate the expression of a serial of defense-related genes in wheat, including *TaMPK3, TaERF3, TaPR1, TaChitinase3, TaChitinase4*, and *Tadefensin*, and positively participated in the pectin-induced resistance responses to *R. cerealis* and *F. pseudograminearum*. This study first revealed that a *WAK*-encoding gene mediated wheat broad resistance to infection of *R. cerealis* and *F. pseudograminearum*.

## Materials and Methods

### Plant and Fungal Materials and Primers

Four wheat (*T. aestivum*) cultivars, namely CI12633, Shanhongmai, Wenmai 6, and Yangmai 9, which exhibit different levels of resistance and susceptibility to sharp eyespot were used to investigate *TaWAK-6D* transcript profiles against *R. ceraelis*. Additionally, six wheat (*T. aestivum*) cultivars, including CI12633, Shanhongmai, Nivat14, Shannong 0431, Yangmai 9, and Yangmai 158, which exhibit different levels of resistance and susceptibility to FCR were used to investigate *TaWAK-6D* transcript profiles against *F. pseudograminearum*. A middle resistant wheat germplasm, CI12633 was used for a VIGS experiment. All wheat seedlings were grown in a greenhouse at 23°C for 14 h in light, and 15°C for 10 h in darkness.

*Rhizoctonia cerealis* strain Rc207 and *F. pseudograminearum* strain WHF220, which were highly virulent in north China, were isolated by Prof. Jinfen Yu and Dr. Li Zhang (Shandong Agricultural University, China). Both of the two fungal strains were cultured on a PDA medium at 25°C in dark conditions.

The sequences of all primers in this study are listed in Supplementary Table 1.

### RNA Extraction and Real-Time Quantitative PCR

Trizol reagent (Invitrogen, USA) was used to extract the total RNA of wheat tissues from different wheat cultivars. Afterward, the RNA was purified and reverse-transcribed into complementary DNA (cDNA) using the FastQuant RT Kit (Tiangen, China) for further reverse transcription PCR (RT-PCR) and real-time quantitative PCR (RT-qPCR) experiments.

In RT-PCR, the transcription level of a barley yellow dwarf virus (BSMV) coat protein (CP) gene was measured to check whether the BSMV was successfully infected with the wheat plants. In RT-qPCR, specific primers were used to measure the transcription level of the *TaWAK-6D, Mitogen-activated protein kinase 3* (*MPK3*), *Ethyleneresponse Factor 3* (*ERF3*), *Defensin, PR1, Chitinase3*, and *Chitinase4* in wheat. RT-qPCR was performed on an ABI7500 instrument (Applied Biosystems, USA) with an SYBR Premix ExTaq kit (TaKaRa, Japan). Reactions were programmed with the following thermal cycling profile: 95°C for 3 min, followed by 40 cycles of 95°C for 5 s, 58°C for 10 s, and 72°C for 32 s. The PCR products were loaded onto 1.5% agarose gels and visualized under UV after staining with ethidium bromide. Each experiment was replicated three times. The relative expression of target genes was calculated using the 2^−ΔΔ*CT*^ method, with the wheat actin gene *TaActin* used as an internal reference gene.

### RNA-Seq Analysis

In this study, the recombinant inbred lines (RILs) derived from the cross Shanhongmai × Wenmai 6, were used for RNA-Seq (RNA-sequencing) analysis as described by Zheng et al. (2020) and Guo et al. (2021).

### Sequence Analyses of *TaWAK-6D*

The full-length open reading frame (ORF) sequence of *TAWAK-6D* was amplified with specific primers *TaWAK-6D*F1/R1 from cDNA of CI12633 plants. The PCR products were cloned into a pMD18-T vector (TaKaRa, Japan) and then sequenced. The predicted protein sequence was analyzed with the Compute pI/Mw tool (http://web.expasy.org/compute_pi/, swiss institute of bioinformatics, Switzerland) to determine the theoretical isoelectric point (pI) and molecular weight (Mw), with the inter Pro-Scan (http://www.ebi.ac.uk/interpro/, the EMBL-European bioinformatics institute, UK) to identify domains, and with the Smart software (http://smart.emblheidelberg.de/, Biobyte GmbH, Germany) to predict conserved motifs. A phylogenetic tree was constructed using a neighbor-joining method implemented in MEGA 6 software (https://www.megasoftware.net/, research center for genomics and bioinformatics, Japan) after alignment with other WAK protein sequences using ClustalW software (https://www.Genome.jp/tools-bin/clustalw, Kyoto university bioinformatics center, Japan).

### Subcellular Localization of TaWAK-6D

The coding region of *TaWAK-6D* lacking the stop codon was amplified using gene-specific primers TaWAK-6D-GFP-inF1/R1. The amplified fragment was digested with restriction enzyme *Bam*H I, then it was subcloned into the 5'-terminus of the green fluorescent protein (GFP) coding region of the pCaMV35S: GFP vector, resulting in the TaWAK-6D-GFP fusion construct pCaMV35S: TaWAK-6D-GFP. The p35S: TaWAK-6D-GFP fusion construct or p35S: GFP control construct was individually introduced into wheat protoplasts (Yoo et al., 2007). The p35S: AtPAI2A-mCherry was used as a plasma marker (PM) in this study, and the PM construct was transfected with p35S: TaWAK-6D-GFP fusion construct into wheat protoplasts. After incubation at 25°C for 16 h, GFP signals were observed and photographed using a confocal laser scanning microscope (Zeiss LSM 700, Germany Carl Zeiss, Germany) with a Fluor × 10**/0.5** M27 objective lens and SP640 filter.

### VIGS and Assessment for Wheat Resistance to *R. cerealis* or *F. pseudograminearum*

Barley yellow dwarf virus-mediated VIGS has been successfully utilized to study gene function in barley and wheat. In this study, a 208 bp fragment of *TaWAK-6D* was subcloned in the antisense orientation into the *Nhe* I restriction site of the RNA γ of BSMV, to form a BSMV: *TaWAK-6D* recombinant construct. Then, the tripartite cDNA chains of BSMV: *TaWAK-6D* or the control BSMV: GFP virus genomes were separately transcribed into RNAs, mixed, and used to infect CI12633 seedlings at the three-leaf stage. At 15 days after virus infection, the fourth leaves of the inoculated seedlings were collected to monitor BSMV infection and to evaluate the transcript change of *TaWAK-6D*. Afterward, the gene-silenced and the BSMV: GFP-infected (control) CI12633 plants were further inoculated with *R. cerealis or F. pseudograminearum*.

*Rhizoctonia cerealis* inoculation and assessment of wheat resistance to *R. cerealis* were performed as previously described by Chen et al. (2008). Briefly, toothpicks were boiled for 30 min, then transferred into flasks for sterilization at 121°C, 0.1 Mpa for 20 min, then placed the toothpicks on PDA medium covered with *R. cerealis* hyphae. After 20 days of darkness culture, these toothpicks (about 3 cm) were covered with the mycelia of *R. cerealis* WK207. Then, the toothpicks harboring well-developed mycelia were embedded into the gap between the leaf sheaths and stems. After inoculation, the stems were wrapped with moist cotton to keep them moist for 4 days. After 35 days inoculation with *R. cerealis*, the infection types (ITs) of these wheat plants were scored from 0 to 5, and the corresponding disease index (DI) was calculated. The disease severity assessments were performed in two independent batches, and each batch included 20 *TaWAK-6D* silenced or BSMV: GFP control wheat seedlings.

Specific identification criteria for sharp eyespot: IT 0 means no disease spots on wheat stems; IT 1 means disease spots only on the leaf sheaths, but no disease spots on the stem; IT 2 means the width of the disease spots on the stems were shorter than the 1/4 circumference of the stem; IT 3 means the width of the disease spots on the stems were longer than 1/4 but shorter than 1/2 circumference of the stems; IT 4 is the width of the disease spots were longer than 1/2 but shorter than 3/4 circumference of the stems; IT 5 means the width of the diseased spots were longer than 3/4 circumference of the stems.

*Fusarium pseudograminearum* inoculation of wheat resistance responses was performed according to Yang X. et al. (2019) with a slight modification. In this study, we used wheat grains instead of millet grains. Briefly, wheat grains were soaked into water for 12 h and transferred into flasks for sterilization at 121°C, 0.1 Mpa for 20 min. Then, a piece of *F. pseudograminearum* hyphae was inoculated into the flask, and cultured at 25°C under darkness for 10 days. For inoculating, 20 g *F. pseudograminearum* colonized wheat grains per flowerpot were weighed, and evenly distributed them near the stem of wheat seedlings, then covered with 50 g sterilized soil. At 30 days post inoculation (dpi) with *F. pseudograminearum*, the ITs of these wheat seedlings were scored from 0 to 5, and the corresponding DI was calculated. The disease severity assessments were performed in two independent batches, and each batch included 20 *TaWAK-6D* silenced or BSMV: GFP control wheat seedlings.

The assessment methods of FCR disease severity were referred to the existing works of research (Liu et al., 2010; Yang et al., 2010; Zheng et al., 2015), and were slightly modified. The specific criteria for identification of FCR are as follows: IT 0 means no disease spots on stems of wheat seedlings; IT 1 means the length of disease spots on wheat stems was <1 cm; IT 2 means the length of the disease spots on wheat stems was between 1 and 2 cm; IT 3 means the lesion length of the disease spots on wheat stems was longer than 2 cm, but the wheat plants were not wilted yet; IT 4 means the wheat plants were wilted, but not death; IT 5 means the wheat plants were dead.

### Ectopic Expression of *TaWAK-6D* in *N. benthamiana* Leaves

The coding region of *TaWAK-6D* was amplified using gene-specific primers pMAS-TaWAK-6D-GFP-inF1/R1. Then, the amplified fragment was digested with restriction enzyme HindIII and subcloned into the 5′-terminus of the GFP coding region in the pMAS: GFP vector, forming the pMAS: TaWAK-6D-GFP fusion construct. Afterward, the pMAS: TaWAK-6D-GFP fusion construct or the pMAS: GFP control construct was separately introduced into *Agrobacterium* strain GV3101 and infiltrated into *N. benthamiana* leaves to transiently express (Hecker et al., 2015). After infiltration, the *N. benthamiana* seedlings were darkly incubated for 24 h at 25°C and cultivated under light for 24 h at 25°C.

Finally, GFP signals were observed using a confocal laser scanning microscope (Zeiss LSM 700, Germany) with a Fluor × 10/0.50 M27 objective lens and SP640 filter. Then, total RNA was extracted from *N. benthamiana* leaves and used for further RT-qPCR analysis.

### Chitin or Pectin Treatment

Before treatment, wheat seedlings were cultured in a greenhouse at 25°C for 10 days. Then chitin or pectin each was suspended in sterile water and used at a final concentration of 100 μg/ml. Subsequently, the wheat seedlings were sprayed with distilled water (mock), pectin solution, or chitin solution, until the surface of all seedlings was covered with a wet solution or water drops. The bottom leaves of the wheat seedlings were collected and RNAs were extracted at 0, 5, 10, and 30 min for further RT-qPCR analysis.

## Results

### *TaWAK-6D* Is Involved in Wheat Resistance Responses to *R. cerealis* and *F. pseudograminearum*

**Figure 1:**
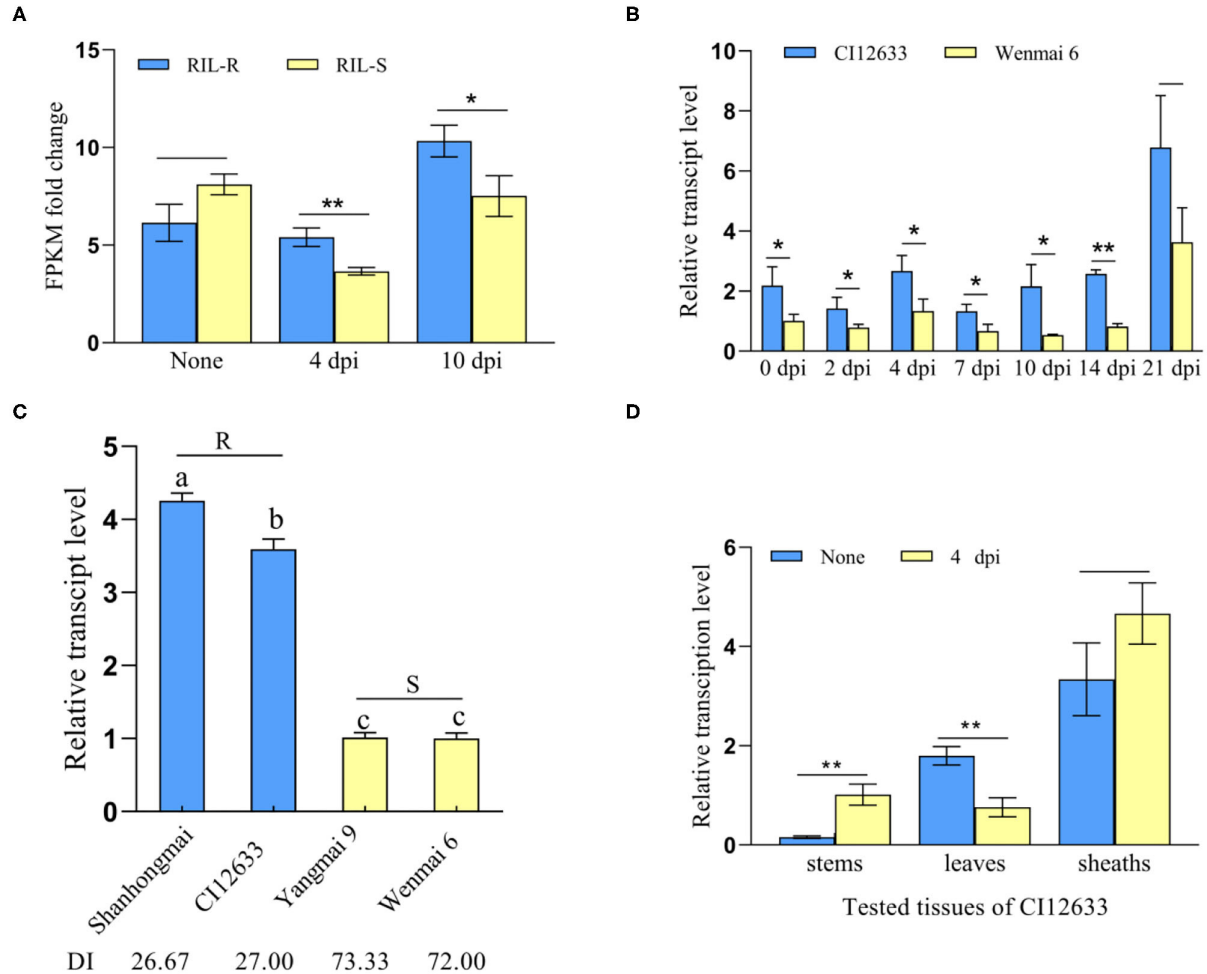
*TaWAK-6D* was a response to *Rhizoctonia cerealis*. **(A)** The transcript pattern of *TaWAK-6D* in the RNA-sequencing (RNA-seq) data. The expression level of *TaWAK-6D* in the resistant recombinant inbred lines (RILs) (RIL-R) was higher than in the susceptible RIL (RIL-S) at 4 and 10 dpi with *R. cerealis*. No treatment means there was no *R. cerealis* inoculated on the seedings. Fragment per kilobase of exon model per million mapped reads (FPKM) was used to indicate the transcript level. **(B)** *TaWAK-6D* transcript in *R. cerealis*-resistant wheat line CI12633 and highly-susceptible wheat cultivar Wenmai 6 at 0, 2, 4, 7, 10, and 14 dpi with *R. cerealis* Rc207. *TaWAK-6D* transcript level at non-treatment was set to 1. **(C)** Transcript patterns of *TaWAK-6D* in four wheat cultivars at 4 dpi with *R. cerealis*. The disease index (DI) means the disease severity after inoculated with *R. cerealis*. The transcript level of *TaWAK-6D* in Wenmai 6 was set to 1. **(D)** Transcript pattern of *TaWAK-6D* in leaves, sheaths, and stems of CI12633 at 4 dpi with *R. cerealis* or non-treatment. *TaActin* was used as an internal control gene (*t*-test: *P < 0.05; **P < 0.01). Bars indicate SEs of the mean.

Based on the RNA-seq data of resistant RILs and susceptible RILs, derived from the cross Shanhongmai × Wenmai 6, we found that after inoculation with *R. cerealis* WK207, the expression of a wheat WAK-encoding gene (sequence ID *TraesCS6D02G210200*), was upregulated in the resistant RILs relative to the susceptible RILs (Figure 1A). Sequence analysis and BlastP (https://blast.ncbi.nlm.nih.gov/Blast.cgi) results indicated that the gene (*TraesCS6D02G210200*) encoded a cell WAKs and was located on wheat chromosome-6D, and the gene, thus, was named as *TaWAK-6D*. Although the transcript level of *TaWAK-6D* in the resistant RILs was only 0.76-fold compared with that in the susceptible RILs at mock inoculation, while after *R. cerealis* inoculation (4 and 10 dpi), the gene transcript was elevated to 1.47- and 1.37-fold in the resistant RILs compared with the susceptible RILs (Figure 1A).

Further RT-qPCR results showed that after inoculation with *R. cerealis, TaWAK-6D* transcript level was significantly greater in resistant wheat cv.CI12633 than the susceptible wheat cultivar Wenmai 6 (Figure 1B), i.e., 2.06- and 4.07-fold higher at 4 and 10 dpi with *R. cerealis*. The analyses were in agreement with the gene transcript trend in the RNA-seq data. Importantly, among the four different wheat cultivars, the *TaWAK-6D* transcript level was the highest in *R. cerealis* resistant wheat cultivar Shanhongmai, followed by the resistant wheat. cv. CI12633, but was the lowest in two highly susceptible cultivars Yangmai 9 and Wenmai 6 (Figure 1C). This result suggested that the transcript abundance of *TaWAK-6D* was corresponding to the resistant degree of the four wheat cultivars. The expression patterns of different tissues showed that at 4 dpi with *R. cerealis*, the higher-level change appeared at the stems of CI12633 seedlings (Figure 1D), where sharp eyespot disease usually occurs. These data suggested that *TaWAK-6D* might participate in wheat resistance responses to *R. cerealis* infection.

**Figure 2:**
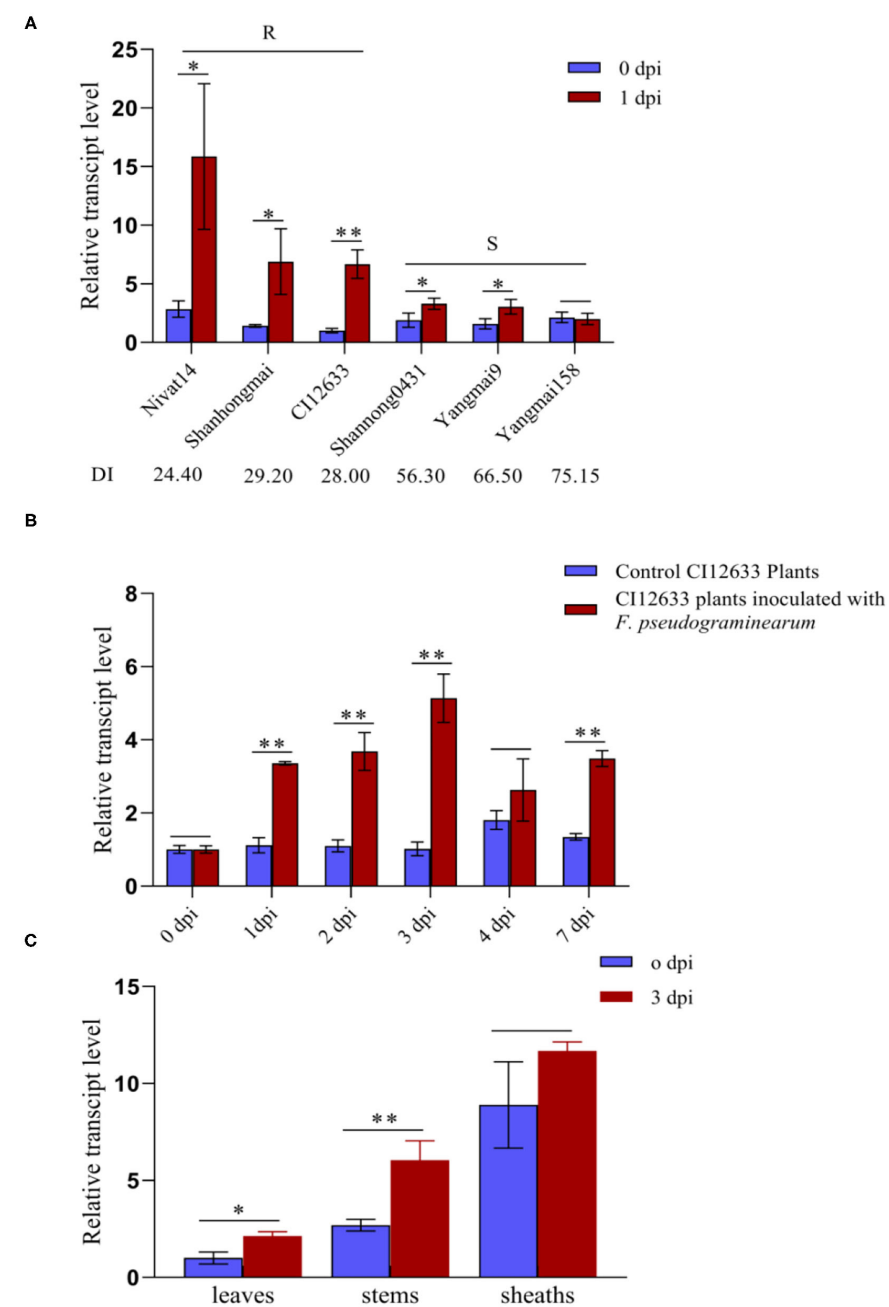
Transcription profiles of *TaWAK-6D* responding to *Fusarium pseudograminearum* in wheat. **(A)** Expression patterns of *TaWAK-6D* in 6 wheat cultivars with different Fusarium crown rot (FCR)-resistance degrees at 0 dpi and 1 dpi with *F. pseudograminearum*. The expression level of *TaWAK-6D* in Yangmai 158 at 0 dpi was set to 1. **(B)** Expression patterns of *TaWAK-6D* in CI12633 plants with *F. pseudograminearum* or in control CI12633 plants at 0, 1, 2, 3, 4, and 7 dpi. *TaWAK-6D* transcript level with 0 dpi was set to 1. **(C)** Expression pattern of *TaWAK-6D* in leaves, stems, and sheaths of CI12633 at 0 dpi or 3 dpi with *F. pseudograminearum*. *TaActin* was used as an internal control gene (*t*-test: *P < 0.05; **P < 0.01). Bars indicate SEs of the mean.

To explore whether *TaWAK-6D* is also involved in the wheat resistance to *F. pseudograminearum*, we deployed RT-qPCR to examine the transcript profiles of *TaWAK-6D* in wheat at 0 and 1 dpi with *F. pseudograminearum* strain WHF220. As shown in Figure 2A, *TaWAK-6D* transcript abundance was significantly raised in the tested wheat cultivars, except the highly susceptible wheat cultivar Yangmai 158, after the pathogen infection, and the induction was higher in FCR-resistant wheat cultivars CI12633, Shanhongmai, and Nivat14 than in the FCR susceptible cultivars (Shannong 0431, Yangmai 9, and Yangmai 158). Additionally, *TaWAK-6D* transcript in the resistant wheat cultivar CI12633 was significantly increased after inoculation with *F. pseudograminearum*, from 1 to 7 dpi, and peaked at 3 dpi (~5.13-fold) compared with the untreated one (Figure 2B). Tissue expression analysis indicated that at 3 dpi with *F. pseudograminearum*, the largest induction appeared on the stems of CI12633 seedlings (Figure 2C), consistent with the majorly-occurring tissue of FCR symptom.

### Sequence and Phylogeny Relationship of TaWAK-6D

**Figure 3:**
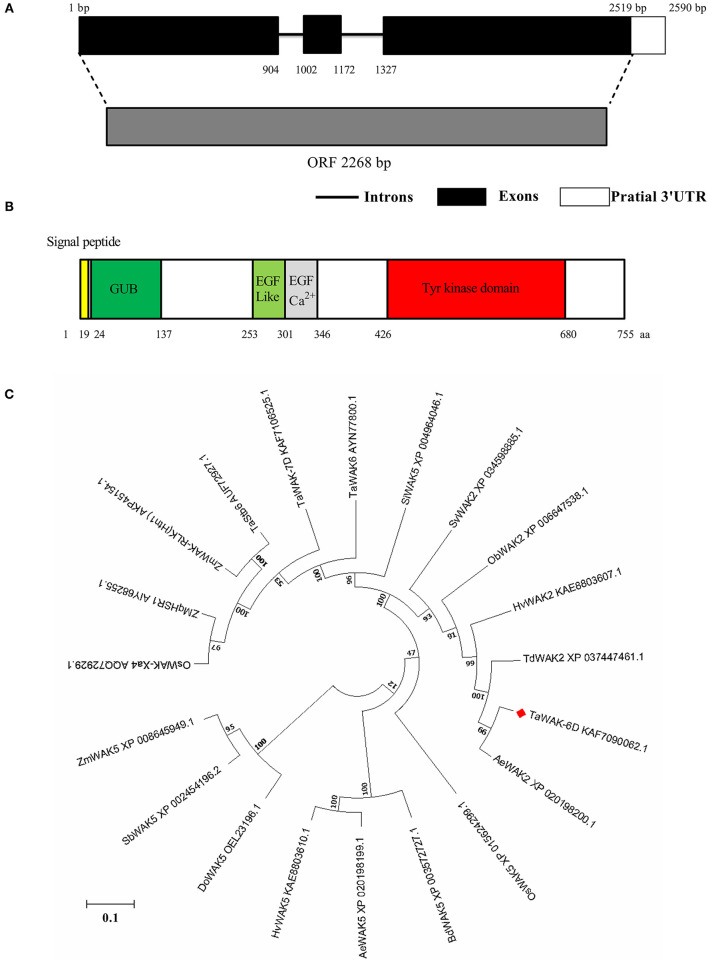
Gene-structure analysis, conserved-domain analysis, and phylogenetic tree construct of TaWAK-6D. **(A)** Gene structure of *TaWAK-6D*. Black boxes represent exons, and black lines indicate introns. **(B)** Schematic diagram of the TaWAK-6D protein. The colored regions indicate different domains. **(C)** Phylogenetic analysis of TaWAK-6D and 19 other WAK proteins. The neighbor-joining method (MEGA 6.0) was used to construct the bootstrapped phylogenetic tree. The position of TaWAK-6D was indicated with a red blot.

The full-length coding sequence of *TaWAK-6D* was applied from the cDNA of the resistant wheat cv. CI12633 seedlings, which is containing an ORF with a 2,268 bp length sequence (Figure 3A). The predicted *TaWAK-6D* protein consisted of 755 amino acid (aa) residues with a predicted Mw of 82.32 kDa and theoretical pI was 6.16. As shown in Figure 3B, the TaWAK-6D protein includes a signal peptide (no. 1–19 aa), an extracellular GUB domain (no. 24–137 aa) that was believed to be closely connected to the cell wall, 1 EGF-like domain (no. 253–301 aa), a calcium-binding EGF domain (EGF-Ca^2+^, no. 302–346 aa), and an intracellular Tyrosine (Tyr) kinase domain (no. 426–695 aa). Through BLAST search against the wheat public genome database (http://plants.ensembl.org/index.html), the results showed that one additional homoeologous gene located on chromosomes 6A was designated as *TaWAK-6A* (*TraesCS6A02G225400*). *TaWAK-6D* and *TaWAK-6A* genomic sequences all contain three exons and two introns. Pairwise comparison of the two homoeologs showed that the protein sequence and coding sequence of *TaWAK-6D* both shared 97% identity with that of *TaWAK-6A* (Supplementary Figures 1, 2), and both proteins contained the same domains, suggesting that both proteins might have redundant functions. However, the transcript level of *TaWAK-6A* in wheat plants was not changed significantly in response to *R. cerealis* (Supplementary Figure 3).

Phylogenetic tree construction, based on the whole protein sequences of *TaWAK-6D* protein and 19 WAKs from various plant species, indicated that WAKs were roughly grouped into three branches (Figure 3C). Almost all WAK5 family members clustered on the same branch, including *Zea, mays ZmWAK5, Sorghum bicolor SbWAK5, Dichanthelium oligosanthes DoWAK5, Hordeum vulgare HvWAK5, Aegilops tauschii AeWAK5, Brachypodium distachyon* BdWAK5, and *Oryza sativa OsWAK5*. The second branch included *Setaria italica* SiWAK5, and six previously reported disease resistance-related WAKs of monocots, including *Zea, mays ZmWAK-qHSR1* (Zuo et al., 2015), *ZmWAK-RLK1* (*Htn1*) (Hurni et al., 2015), *O. sativa OsWAK-Xa4* (Hu et al., 2017), *T. aestivum* Stb6 (Saintenac et al., 2018), *TaWAK-7D* (Qi et al., 2021), and *TaWAK6* (Dmochowska-Boguta et al., 2020). Meanwhile, *T. aestivum TaWAK-6D* and other five WAK2 family members were clustered on the third branch, including *Setaria viridis Sv*WAK2, *Oryza brachyantha ObWAK2, H. vulgare HvWAK2, Triticum dicoccoides TdWAK2*, and *A. tauschii* subsp. *AeWAK2*. Further sequence analysis showed that the *TaWAK-6D* protein sequence is identical (100% identity) to *A. tauschii* WAK2 *AeWAK2*, suggesting that *TaWAK-6D* might origin from *AeWAK2*.

### *TaWAK-6D* Localizes at the Plasma Membrane in Wheat

**Figure 4:**
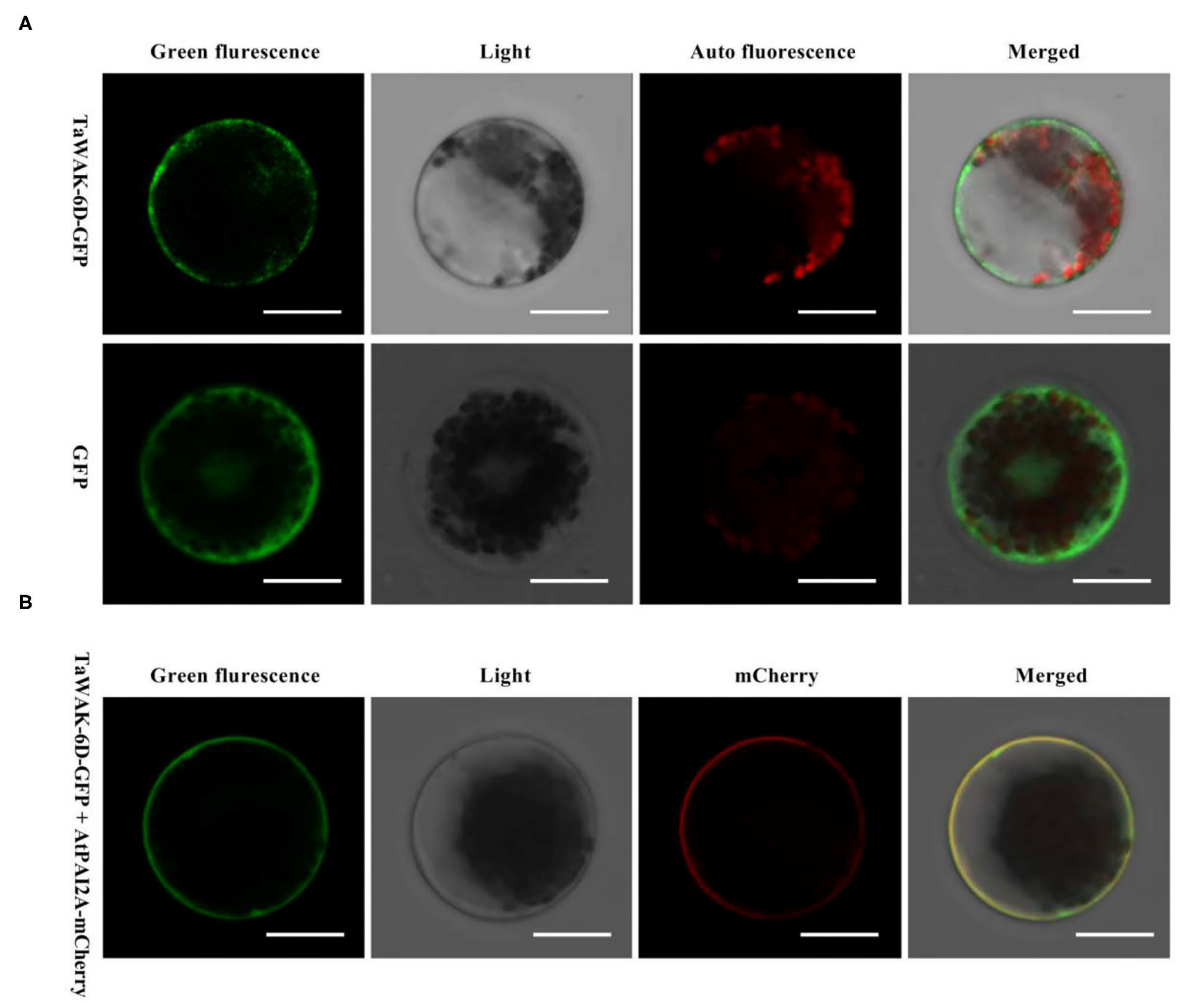
Subcellular localization of TaWAK-6D protein in wheat protoplasts. **(A)** The control 35S: GFP and fused 35S: TaWAK-6D–GFP construct plasmids were transiently expressed in mesophyll protoplasts cells. The red color was auto-fluorescence from the wheat chloroplast. **(B)** The fused 35S: TaWAK-6D–GFP and the plasma marker (PM) 35S: AtPAI2A–mCherry vector plasmids were co-transiently expressed in mesophyll protoplasts cells. Scale bars = 20 μm.

To investigate the subcellular localization of *TaWAK-6D* in wheat plant cells, we prepared the p35S: *TaWAK-6D*-GFP fusion construct. The resulting fusion construct and the control p35S: GFP were separately introduced into the wheat mesophyll protoplasts and then transiently expressed at 25°C under darkness for 16 h. As shown in Figure 4, the *TaWAK-6D*-GFP protein is distributed at the plasma membrane with the membrane-localized marker in wheat protoplasts. In contrast, GFP was expressed in all parts of protoplasts. These results suggested that *TaWAK-6D* localizes at the plasma membrane in wheat.

### Silencing of *TaWAK-6D* Impairs Wheat Resistance Both to *F. pseudograminearum* and *R. cerealis*

**Figure 5:**
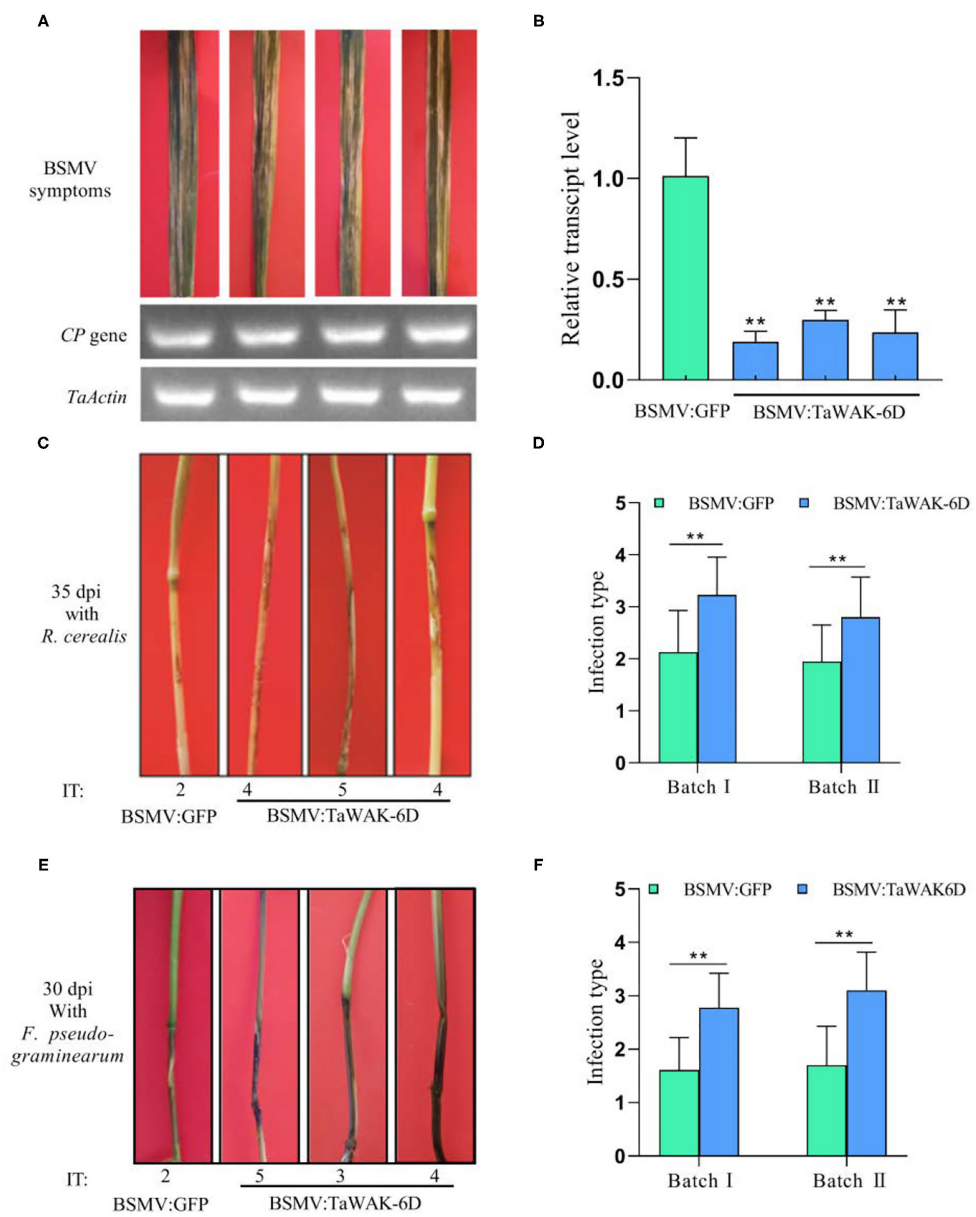
Silencing of *TaWAK-*6D increases wheat susceptibility to *R. cerealis* and *F. pseudograminearum*. **(A)** The typical symptom of BSMV on wheat leaves after being infected by BSMV: GFP or BSMV: *TaWAK-6D* for 15 days. A BSMV coat protein (CP) was used as a marker gene to detect the BSMV. **(B)** The transcript level of *TaWAK-6D* in *TaWAK-6D*-silencing and BSMV: GFP-infected wheat CI12633 plants. The transcript level of *TaWAK-6D* in BSMV: GFP- infected wheat CI12633 plants was set to 1. Significant differences were determined based on three technical repeats (*t*-test: **P < 0.01). **(C)** Sharp eyespot symptoms on *TaWAK-6D* silencing and control CI12633 plants at 35 dpi with *R. cerealis*. Disease severity was indicated by infection type (IT). **(D)** Mean IT of *TaWAK-6D* silencing or control CI12633 plants at 35 dpi with *R. cerealis* in two independent batches (*t*-test: **P < 0.01). Bars indicate SEs of the mean. **(E)** FCR symptoms of BSMV: *TaWAK-6D* or BSMV: GFP CI12633 plants after inoculated with *F. pseudograminearum* for 30 days. The IT was used to indicate the disease severity of each seedling **(F)** Mean ITs of BSMV: *TaWAK-6D or* BSMV: GFP CI12633 control seedlings at 30 dpi with *F. pseudograminearum* in two independent batches (*t*-test: **P < 0.01). Bars indicate SEs of the mean.

Barley yellow dwarf VIGS was used to investigate the defense role of *TaWAK-6D* in wheat against fungal diseases. First, a 208 bp fragment (locating on 2,383–2,590 bp) of *TaWAK-6D* was selected by si-Fi software, and then the fragment was sub-cloned in the antisense orientation into the RNA γ of BSMV to form a BSMV: *TaWAK-6D* construct (Supplementary Figures 4, 5). Then, the resulted BSMV: *TaWAK-6D* recombinant virus and the control BSMV: GFP virus was transfected into leaves of the resistant wheat cultivar CI12633. At 15 days post the transfection, the BSMV infection symptom appeared in the newly emerged leaves and the transcript of BSMV CP was detected (Figure 5A), indicating that the BSMV had infected these wheat plants. Meanwhile, RT-qPCR results showed that, compared with the BSMV: GFP -infected CI12633 plants, the transcript level of *TaWAK-6D* was significantly lower in BSMV: *TaWAK-6D* infected CI12633 plants (Figure 5B), indicated *TaWAK-6D* was successfully silenced.

Furthermore, these *TaWAK-6D-*silenced and control wheat plants were individually inoculated with either *R. cerealis* strain WK207 or *F. pseudograminearum* strain WHF220. At 35 dpi with *R. cerealis* WK207, compared with BSMV: GFP-infected CI12633 plants, *TaWAK-6D*-silenced CI12633 plants displayed more disease severity of sharp eyespot, including larger necrotic areas, and bigger ITs (Figure 5C). The disease scoring results showed that in two VIGS batches the average ITs of *TaWAK-6D*-silenced CI12633 plants were 2.8 and 3.2, and the corresponding average DI were 56 and 64.6, respectively, whereas the average ITs of BSMV: GFP control CI12633 seedlings were 1.9–2.1, and the average DI were 38.9 and 42.5, respectively (Figure 5D, Supplementary Figure 6A). The results showed that expressed *TaWAK-6D* is required for wheat resistance to *R. cerealis*.

Additionally, at ~30 dpi with *F. pseudograminearum*, compared with BSMV: GFP-infected CI12633 plants, the stems of *TaWAK-6D*-silenced CI12633 plants exhibited more disease severity of FCR, including larger necrotic areas and more serious ITs (Figure 5E). The disease tests in two VIGS batches showed that the average ITs of BSMV: GFP-treated CI12633 plants were only 1.6 and 1.7, and the corresponding average DI were 32.2 and 34, whereas the average ITs of *TaWAK-6D*-silenced CI12633 plants were 2.7 and 3.1, and the average DI were 55.5 and 62, respectively (Figure 5F, Supplementary Figure 6B). These data suggested that the expressed *TaWAK-6D* is required for wheat resistance to *F. pseudograminearum*.

### *TaWAK-6D* Is Required for the Expression of Defense-Related Genes

**Figure 6:**
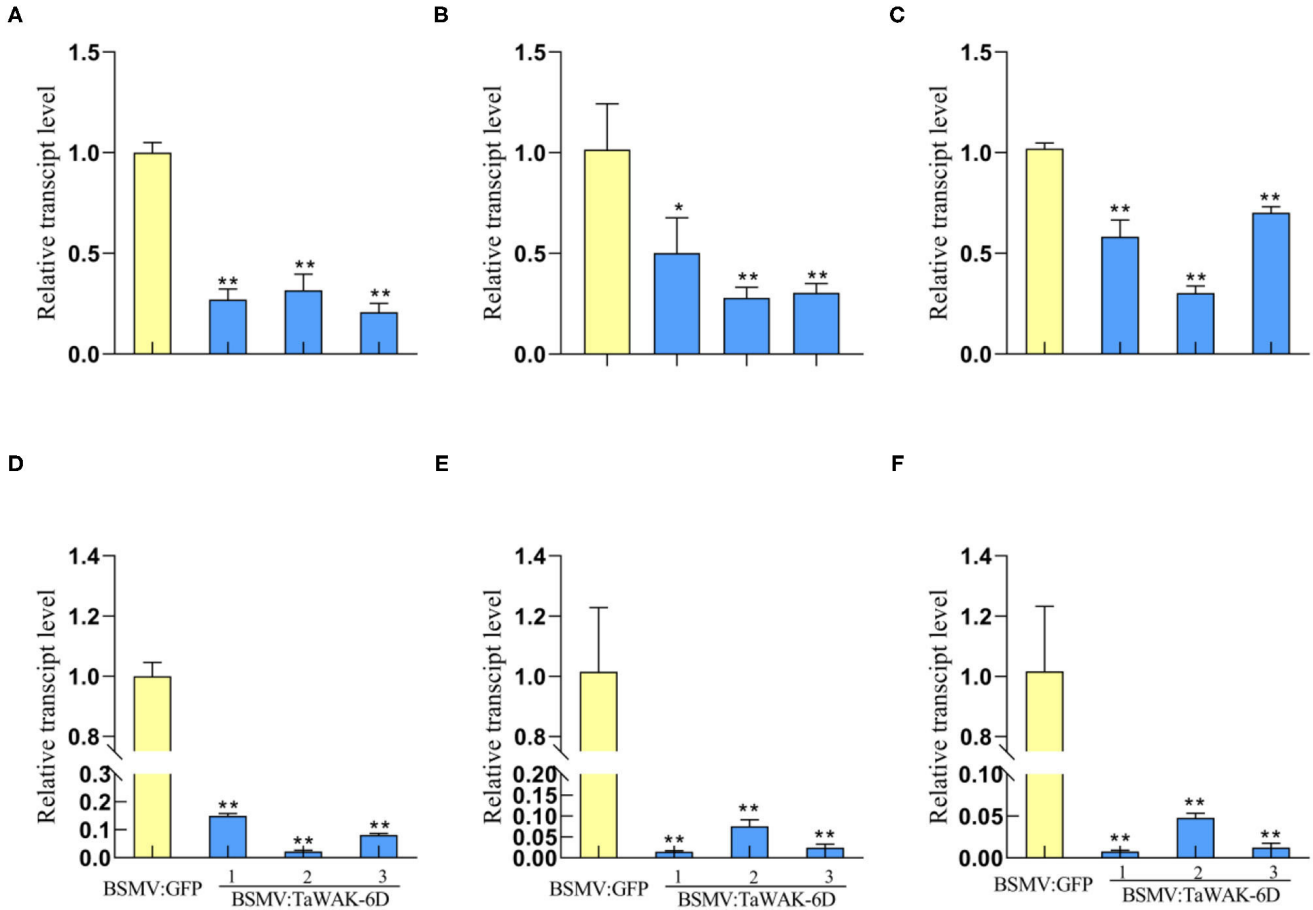
Transcript patterns of defense-related genes in BSMV: GFP-infected and BSMV: *TaWAK-6D*-silenced wheat plants. Relative transcript abundances of *TaMPK3* **(A)**, *TaERF3* **(B)**, *TaPR1* **(C)**, *TaDefensin* **(D)**, *TaChitinase3* **(E)**, and *TaChitinase4* **(F)** in BSMV: *TaWAK-6D*-silenced CI12633 plants were quantified relative to those in BSMV: GFP-infected control plant. All samples were taken from sheaths of wheat plants. Statistically significant differences between BSMV: *TaWAK-6D*-silenced and BSMV: GFP-infected wheat plants were determined based on three replications using a *t*-test (*P < 0.05; **P < 0.01). Bars indicate the SEs of the mean. *TaActin* was used as an internal control.

To investigate whether *TaWAK-6D* is required for the expression of defense-related genes in wheat, RT-qPCR was used to examine the transcript levels of wheat defense-related genes in *TaWAK-6D*-silenced and the BSMV: GFP-infected (control) wheat plants. The tested genes included *TaMPK3, TaERF3, Tadefensin, TaPR1, TaChitinase3*, and *TaChitinase4*, which have been shown to participate positively in wheat resistance responses to fungal diseases (Zhu et al., 2017; Wang K. et al., 2020). The analyses showed that the transcript levels of these six genes were significantly decreased in *TaWAK-6D*-silenced CI12633 plants relative to the BSMV: GFP-infected CI12633 plants (Figures 6A–F). These results suggested that the silencing of *TaWAK-6D* represses the expression of these six defense-associated genes in wheat.

### *TaWAK-6D* Positively Modulates the Expression of Defense-Related Genes

**Figure 7:**
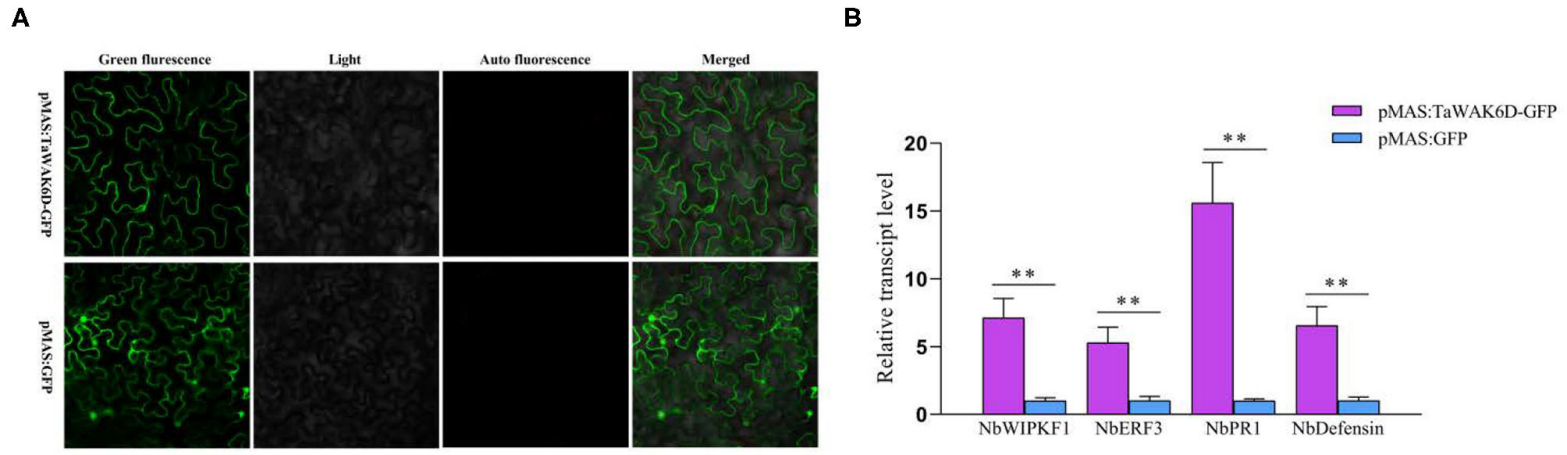
Transcript patterns of defense-related genes in pMAS: GFP and pMAS: TaWAK-6D-GFP ectopic expression *Nicotiana benthamiana* leaves. **(A)** Ectopic expression of *TaWAK-6D* in *N. benthamiana* leaves. a pMAS: TaWAK-6D-GFP fusion construct and the control pMAS: GFP vectors were separately introduced into *Agrobacterium* and infiltrated into *N. benthamiana* leaves to transiently expressed for 48 h. **(B)** The transcript level of defense-related genes in pMAS: TaWAK-6D-GFP and the pMAS: GFP (control) ectopic expression *N. benthamiana* leaves. Statistically significant differences between pMAS: TaWAK-6D-GFP and the pMAS: GFP (control) *N. benthamiana* leaves were determined based on three replications using a *t*-test (**P < 0.01). Bars indicate the SEs of the mean. *NbActin* was used as an internal control.

To investigate whether *TaWAK-6D* can positively modulate the expression of defense-related genes, a pMAS: *TaWAK-6D*-GFP fusion vector and the control pMAS: GFP vectors were separately introduced into *Agrobacterium* and infiltrated into *N. benthamiana* leaves to transiently express (Figure 7A). Then, RT-qPCR was used to examine the transcript level of defense-related genes in pMAS: TaWAK-6D-GFP and the pMAS: GFP (control) ectopic expression *N. benthamiana* leaves. The tested genes included *NbWIPK* (homolog gene of *TaMPK3*), *NbERF3, Nbdefensin*, and *NbPR1*. The analyses showed that the transcript levels of four *N. benthamiana* defense-associated genes were significantly increased in pMAS: TaWAK-6D-GFP leaves relative to the pMAS: GFP *N. benthamiana* leaves (Figure 7B). These results indicated that *TaWAK-6D* can positively modulate the expression of several defense-related genes in *N. benthamian*a leaves, and it also implied that *TaWAK-6D* may have similar functions in wheat plants.

### *TaWAK-6D* Is Involved in Pectin-Induced Immune Responses

**Figure 8:**
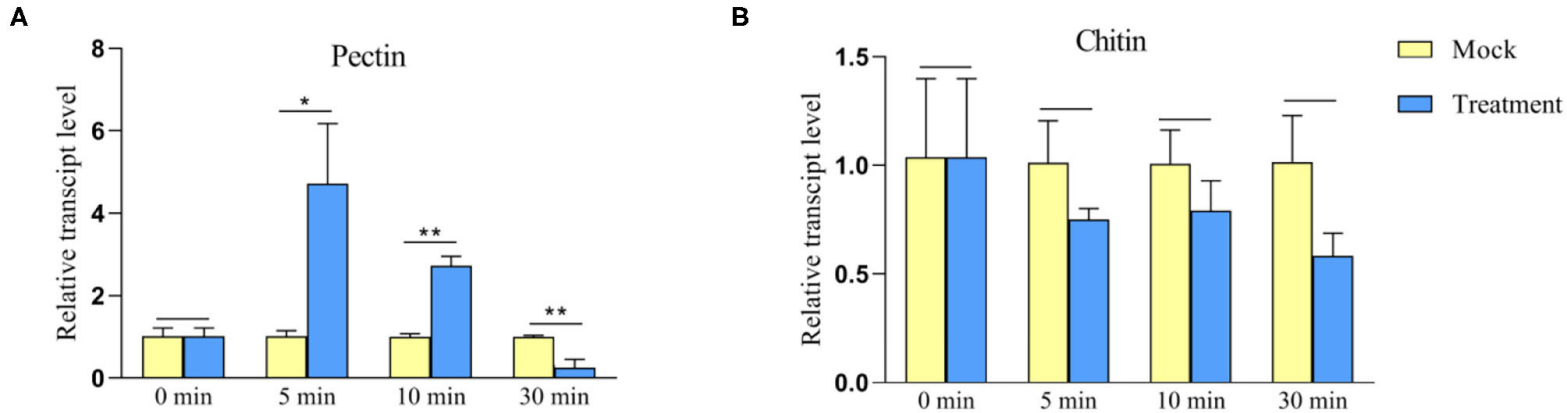
Transcript profiles of *TaWAK-6D* in the wheat bottom leaves treated by either exogenous pectin or chitin. **(A)** Transcript profiles of *TaWAK-6D* in wheat cv. CI12633 leaves treated by 100 μg/ml exogenous pectin. **(B)** Transcript profiles of *TaWAK-6D* in leaves of wheat cv. CI12633 after exogenous application of 100 μg/ml chitin. The transcript level of *TaWAK-6D* in mock-treated wheat plants is set to 1. Statistically significant differences are analyzed based on three replications using a *t*-test (*P < 0.05, **P < 0.01). Bars indicate the SEs of the mean.

Chitin and pectin are conserved components of fungal cell walls, which might trigger plant innate immune responses. To investigate whether *TaWAK-6D* responds to exogenous pectin and chitin stimuli, we analyzed the transcriptional profiles of *TaWAK-6D* in wheat cv. CI12633 treated with either pectin (100 μg/ml) or chitin (100 μg/ml), or mock-solution for 0, 5, 10, and 30 min. As shown in Figure 8, the *TaWAK-6D* transcript level was significantly and rapidly elevated by exogenous pectin treatment but not by chitin treatment, compared with the mock treatment. These data suggested that *TaWAK-6D* might be involved in pectin-induced innate immune responses in wheat.

## Discussion

Some WAK proteins play an important role in disease resistance or mediating defense responses to pathogens (Zuo et al., 2015; Hu et al., 2017; Saintenac et al., 2018). For example, the study of Wang P. et al. (2020) used RNA-seq, RT-qPCR, and VIGS assays to identify the cotton GhWAK7A involved in the resistance response to fungal pathogens *Verticillium dahliae* and *Fusarium oxysporum f. sp vasinfectum*: GhWAK7A showed enhanced expression at 1 dpi, and GhWAK7A-silenced (VIGS) plants were more susceptible to *Verticillium* and *Fusarium* wilt pathogens (Wang P. et al., 2020). In this study, we deployed a set of RNA-Seq data and RT-qPCR analyses and identified the wheat WAK-encoding gene *TaWAK-6D* in wheat resistance responses to both wheat fungal pathogens *R. cerealis* or *F. pseudograminearum*. *TaWAK-6D* transcript abundance was significantly raised after infection of either *R. cerealis* or *F. pseudograminearum*. Interestingly, the transcriptional induction was stronger in the tested resistance wheat genotypes compared with the susceptible wheat genotypes, suggesting that the resistance in wheat genotypes is associated with elevated *TaWAK-6D* expression. As expected, VIGS-based functional assays showed that knock-down of *TaWAK-6D* transcript compromised the resistance of wheat to both sharp eyespots caused by *R. cerealis* and FCR caused by *F. pseudograminearum*. Taken together, these results revealed that *TaWAK-6D* plays a positive role in wheat resistance to both *R. cerealis* and *F. pseudograminearum*, implying a potentially conserved mechanism in wheat responses to both two soil-borne fungal pathogens. The current research broadened our understanding of plant WAKs in plant innate immune responses to pathogens.

The predicted TaWAK-6D protein sequence includes a GUB domain, an EGF-like domain, an EGF-Ca^2+^ domain, a TM region, and a non-RD Tyr kinase domain. A non-RD kinase domain usually was found in plant innate immune receptors (Dardick et al., 2012). For instance, the protein sequences of *ZmWAK-RLK1* encoded by *Htn1, ZmqHSR-*encoding *ZmWAK*, the rice *WAK-Xa4*, and wheat *TaWAK6* for adult resistance to leaf rust all contain a non-RD kinase domain (Hurni et al., 2015; Zuo et al., 2015; Hu et al., 2017; Dmochowska-Boguta et al., 2020). Since the *TaWAK-6D* protein sequence is identical (100% identity) with that of AeWAK2, we supposed that *TaWAK-6D* might originate from *AeWAK2*. In previous studies, a few WAKs have been localized to the plasma membrane in rice *OsWAK1* (Li et al., 2009), *OsDEES1* (Wang et al., 2012), *OsWAK91, OsWAK92*, and *OsWAK14* (Cayrol et al., 2016). More recently, both Maize resistance WAKs, *ZmqHSR1*, and *ZmWAK-Hnt1*, were shown to be localized to the plasma membrane (Hurni et al., 2015; Zuo et al., 2015; Yang et al., 2019). As expected, our assays showed that *TaWAK-6D* localized to the plasma membrane, in line with the immune roles of WAKs on the plasma membrane.

The pectin, OGs, and chitin are common to all fungi, can act as important elicitors in several plant-pathogen interactions (Decreux and Messiaen, 2005; Kohorn et al., 2009). In *Arabidopsis, AtWAK1* and *AtWAK2* were shown to bind pectin and OGs (Decreux and Messiaen, 2005; Kohorn et al., 2009; Brutus et al., 2010). Upon pectin treatment, *AtWAK2* activates the mitogen-activated kinases MPK3 and MPK6 and triggers the innate immune signal transduction of the plant (Kohorn et al., 2009, 2012). A previous study showed that chitin triggers the expression of the rice *OsWAK91/92/14* genes (Delteil et al., 2016). In cotton, *GbWAK7A*, by complex with the chitin sensory receptors, contributes to chitin-induced immune responses (Wang P. et al., 2020). In the current research, pectin, but not chitin, rapidly induced the expression of *TaWAK-6D*, suggesting that *TaWAK-6D* might be involved in pectin-triggering immune responses.

Some RLK proteins at the plasma membrane were shown to be responsible for triggering a cascade of intracellular events during plant innate immune responses. For example, the silencing of *GhWAK7A* compromised activation of *GhMPK6* and *GhMPK3* in cotton, and the expression level of both *GhMPK3* and *GhWRKY30* after chitin treatment was reduced in *GhWAK7A*-silenced cotton plants relative to control plants (Wang P. et al., 2020). In rice, overexpressing *OsWAK25* upregulated the expression of *PR10* and *PBZ1*, in turn, enhanced resistance to the hemibiotrophic pathogens *X. oryzae* and *M. oryzae* (Harkenrider et al., 2016). The maize smut resistance gene *ZmqHSR1* (*ZmWAK*) elevated the expression of *ZmPR-1* and *ZmPR5* after the pathogen infection (Zuo et al., 2015). Previous studies indicated that heightened expression of *TaERF3, Tachitinases*, and *Tadefensin* contributed to the resistance of wheat to sharp eyespot caused by *R. cerealis* (Zhu et al., 2017; Liu et al., 2020). In the current experiments, silencing *TaWAK-6D* not only decreased the expression levels of *TaMPK3, TaERF3, TaPR1, TaChitinase3, TaChitinase4*, and *Tadefensin* but also compromised the resistance of wheat to both *R. cerealis* and *F. pseudograminearum*. Additionally, when ectopic expression *TaWAK-6D in N. benthamiana* leaves, the transcript level of four *N. benthamiana* defense-associated genes was also significantly increased, indicating that the expression of *TaWAK-6D* and these defense-associated genes tested positively participated in wheat defense against invasion of both fungal pathogens.

## Conclusion

In the current research, we identified the wheat WAK-encoding gene *TaWAK-6D* in the plant innate immune responses to both *R. cerealis* and *F. pseudograminearum* at the seedling stage. *TaWAK-6D* transcript abundance was induced after both fungal pathogens and pectin treatment. The expressed *TaWAK-6D* positively regulated the expression of a serial of defense-related genes, including *TaMPK3, TaERF3, TaPR1, TaChitinase3, TaChitinase4*, and *Tadefensin*, and consequently, positively contributed to wheat resistance to sharp eyespot caused by *R. cerealis* and FCR caused by *F. pseudograminearum*. This study provided novel insights into potentially conserved roles of WAKs in wheat resistance responses to fungal pathogens. *TaWAK-6D* is a candidate gene for improving wheat resistance to both important diseases at the seedling stage.

## Data Availability Statement

The original contributions presented in the study are included in the article/Supplementary Material, further inquiries can be directed to the corresponding author/s.

## Author Contributions

ZZ designed the research, supervised the work, revised, and edited the manuscript. HQ, FG, and LL performed the majority of the experiments, analyzed the data, and wrote the draft manuscript. XZ and XW extracted RNAs of RILs and tested disease degree of these wheat cultivars. JY and LZ planted and assessed these wheat materials. All authors contributed to the article and approved the submitted version.

## Funding

This study was funded by the NSFC program (Grant No. 31771789) and National Key Project for Research on Transgenic Biology, China (2016ZX08002001 to ZZ).

## Conflict of Interest

The authors declare that the research was conducted in the absence of any commercial or financial relationships that could be construed as a potential conflict of interest.

## Publisher's Note

All claims expressed in this article are solely those of the authors and do not necessarily represent those of their affiliated organizations, or those of the publisher, the editors and the reviewers. Any product that may be evaluated in this article, or claim that may be made by its manufacturer, is not guaranteed or endorsed by the publisher.

## Supplementary Material

Supplementary Figure 1 Amino acid (aa) sequence alignment of TaWAK-6D and TaWAK-6A.

Supplementary Figure 2 Coding sequence alignment of *TaWAK-6D* and *TaWAK-6A*.

Supplementary Figure 3 The transcript pattern of *TaWAK-6A* in the RNA-seq data. The transcript level of *TaWAK-6A* was not significantly changed after being inoculated with *R. cerealis* in wheat. Statistically significant differences are analyzed based on three replications using a *t*-test. Bars indicate the standard errors of the mean.

Supplementary Figure 4 Off-target prediction by si-Fi software and illustration of recombinant BSMV: *TaWAK-6D*. **(A)** *TaWAK-6D* fragment used for BSMV: *TaWAK-6D* vector construction and selected by an off-target prediction by si-Fi software. **(B)** Illustration of recombinant BSMV: *TaWAK-6D* vector. The orientation of the *TaWAK-6D* insert is indicated by the dark box.

Supplementary Figure 5 Nucleic acid alignment of *TaWAK-6D* VIGS fragment between *TaWAK-6A* in the same position.

Supplementary Figure 6 Comparison of *Fusarium* crown rot or sharp eyespot disease index (DI) between BSMV: GFP or BSMV: *TaWAK-6D* wheat plants. **(A)** Sharp eyespot DI of BSMV: GFP or BSMV: *TaWAK-6D* at 35 dpi with *R. cerealis* in two independent batches. (*t*-test: ^**^*P* < 0.01). Bars indicate SEs of the mean. **(B)** *Fusarium* crown rot DI of BSMV: GFP or BSMV: *TaWAK-6D* at 30 dpi with *F. pseudograminearum* in two independent batches. (*t*-test: ^**^*P* < 0.01). Bars indicate SEs of the mean.

Supplementary Table 1 Primers and their sequences used in this study.
