# Supplementary figures and images for: The Wheat Wall-Associated Receptor-Like Kinase TaWAK-6D Mediates Broad Resistance to Two Fungal Pathogens Fusarium pseudograminearum and Rhizoctonia cerealis
Source: Front Plant Sci. 2021 Oct 27;12:758196. doi: 10.3389/fpls.2021.758196 (PMC8579037; doi:10.3389/fpls.2021.758196)

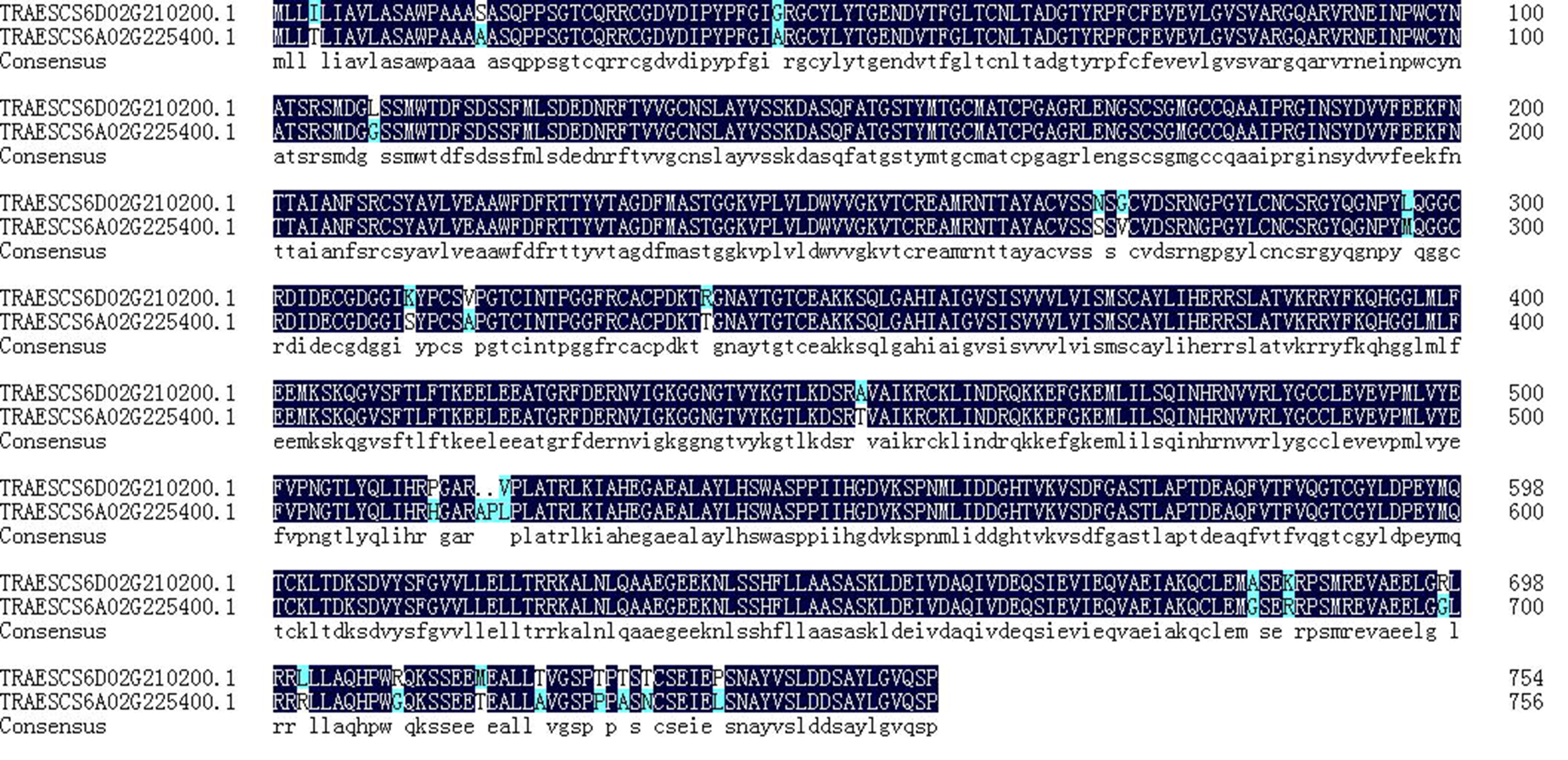

Supplement: Supplementary Figure 1 — Amino acid (aa) sequence alignment of TaWAK-6D and TaWAK-6A. [file Image_1.jpg]

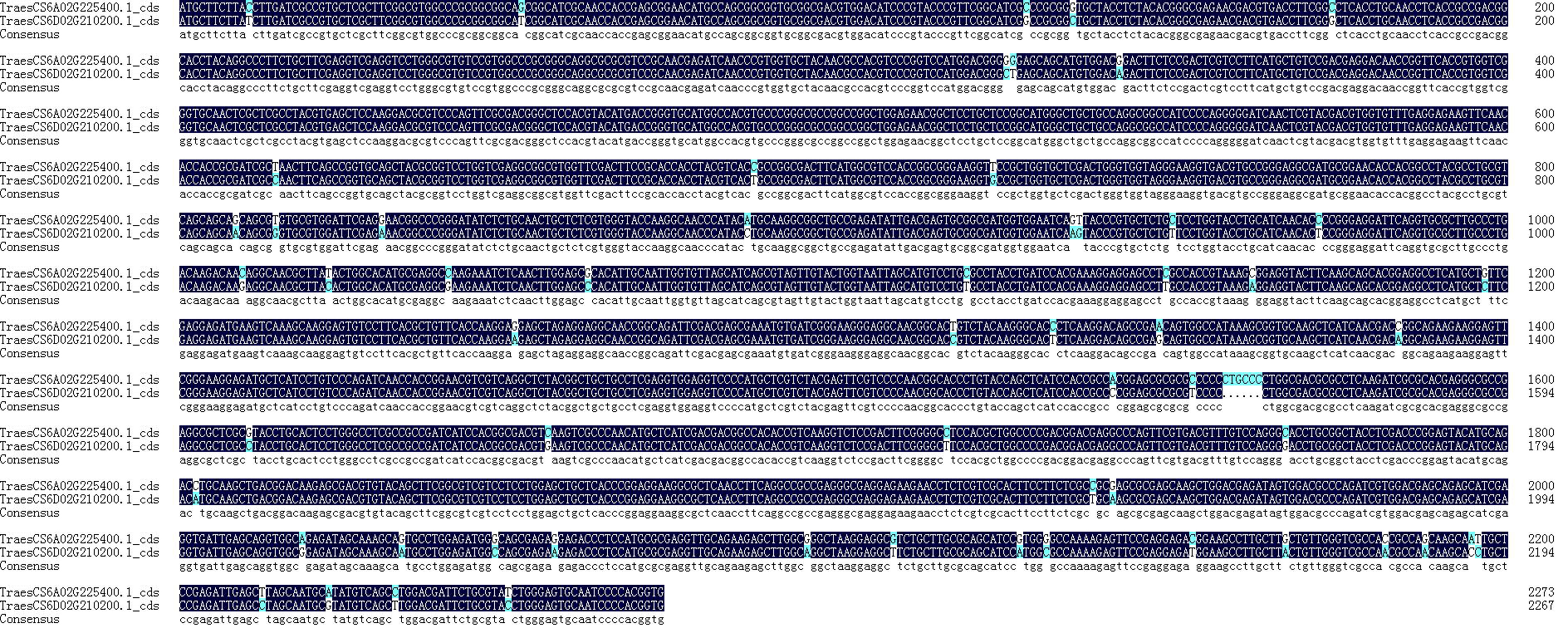

Supplement: Supplementary Figure 2 — Coding sequence alignment of TaWAK-6D and TaWAK-6A. [file Image_2.jpg]

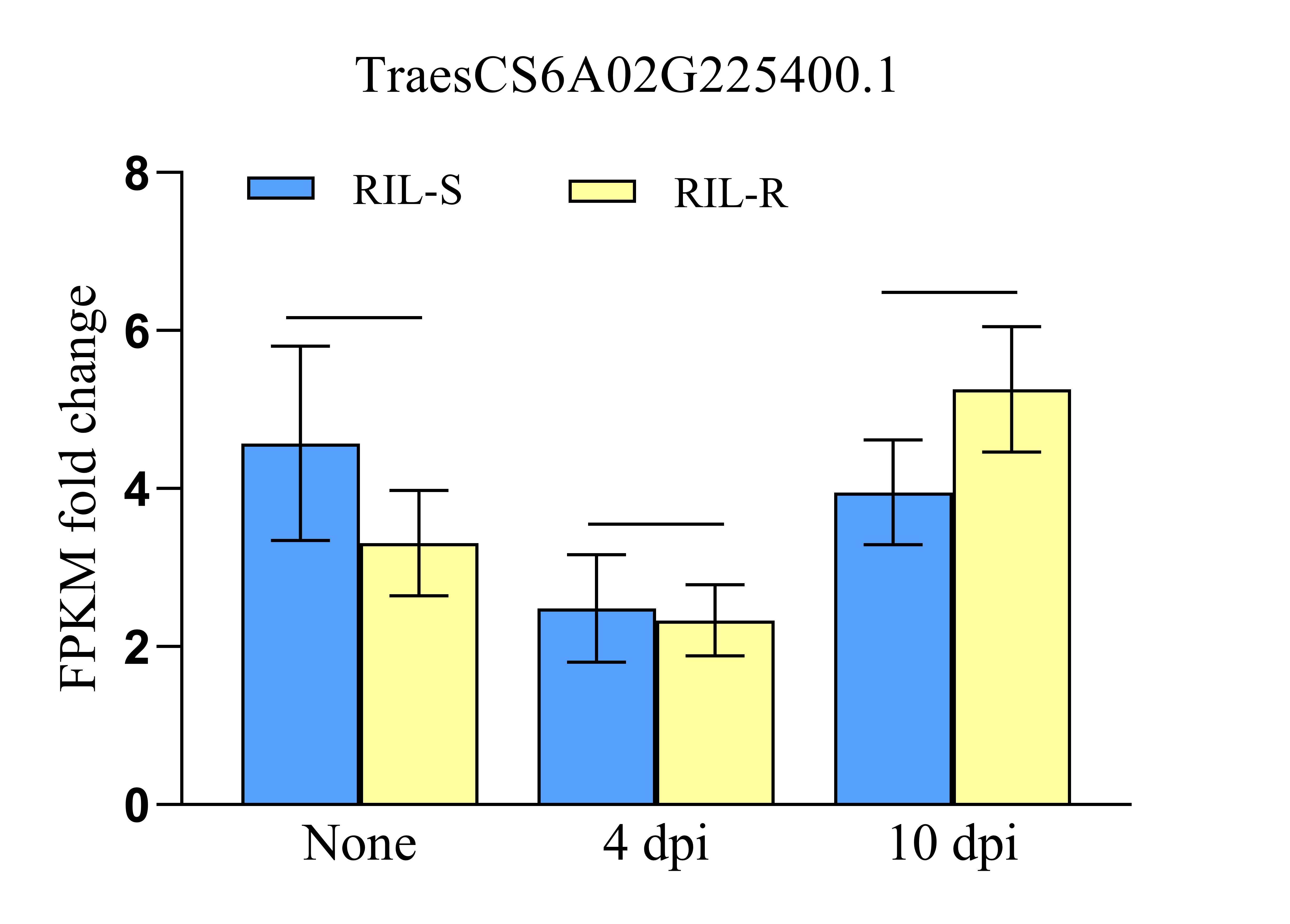

Supplement: Supplementary Figure 3 — The transcript pattern of TaWAK-6A in the RNA-seq data. The transcript level of TaWAK-6A was not significantly changed after being inoculated with R. cerealis in wheat. Statistically significant differences are analyzed based on three replications using a t-test. Bars indicate the standard errors of the mean. [file Image_3.tif]

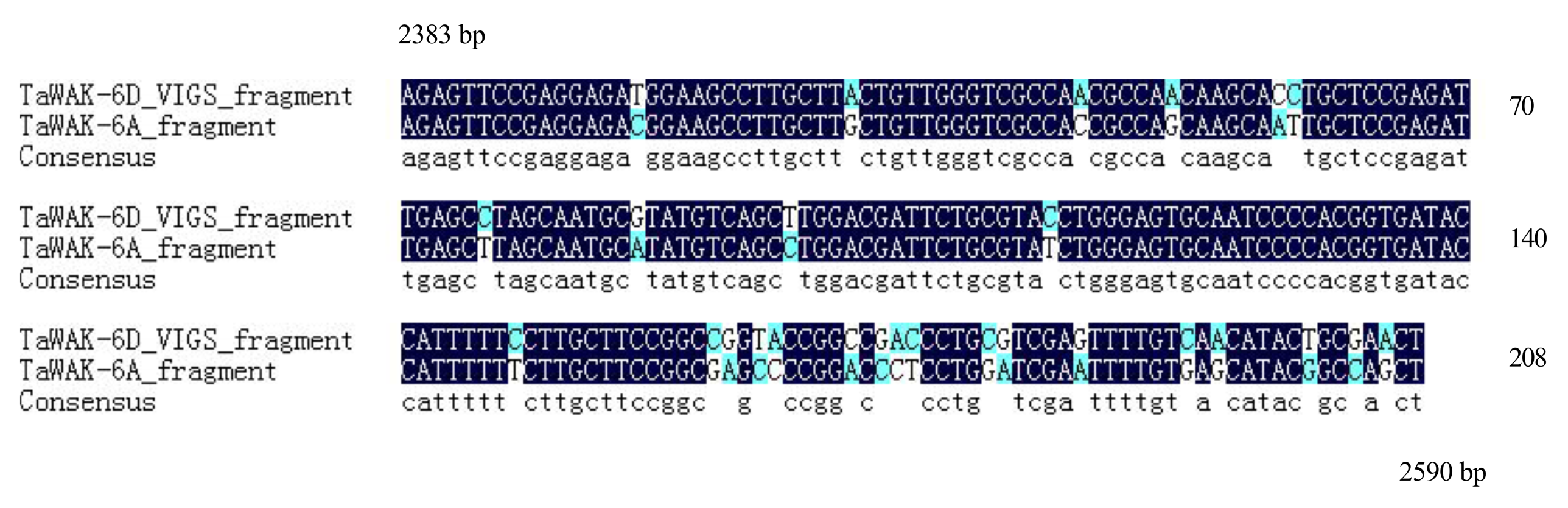

Supplement: Supplementary Figure 5 — Nucleic acid alignment of TaWAK-6D VIGS fragment between TaWAK-6A in the same position. [file Image_5.tif]
