# Supplementary material for: The Wheat Wall-Associated Receptor-Like Kinase TaWAK-6D Mediates Broad Resistance to Two Fungal Pathogens Fusarium pseudograminearum and Rhizoctonia cerealis
Source: Front Plant Sci. 2021 Oct 27;12:758196. doi: 10.3389/fpls.2021.758196 (PMC8579037; doi:10.3389/fpls.2021.758196)
Supplement: Supplementary Table 1 — Primers and their sequences used in this study. [file Table_1.DOCX]

**Table S1** Primers and their sequences used in this study

| **Primer name** | **Sequence (5´–3´ )** | | **Use** |
| --- | --- | --- | --- |
| **Ta6D200-F1** | **5'- AAGGTGTTGTATCCAGAGC -3'** | **PCR for cDNA amplification** | |
| **Ta6D200-R1** | **5'-GGACTACACAAGCACAAG-3'** | **PCR for cDNA amplification** | |
| **Ta6D200-VIGSF1** | **5'-TACGCTAGCCAGAGTTCCGAGGAGATGG-3'** | **VIGS** | |
| **Ta6D200-VIGSR1** | **5'-GACGCTAGCCAGTTCGCAGTATGTTGAC-3'** | **VIGS** | |
| **GFP-6D200-inF** | **5'-TATCTCTAGAGGATCCATGCTTCTTATCTTGATCG-3'** | **subcellular localization** | |
| **GFP-6D200-inR1** | **5'-TGCTCACCATGGATCCCCGTGGGGATTGCACTCC-3'** | **subcellular localization** | |
| **pMAS-TaWAK-6D-GFP-F1** | **5'-CAAATCGACTCTAGAAAGCTTATGCTTCTTATCTTGATCGC-3'** | **ectopic expression** | |
| **pMAS-TaWAK-6D-GFP-R1** | **5'-TGCTCACCATCTGCAGAAGCTTCCGTGGGGATTGCACTCC-3'** | **ectopic expression** | |
| **Ta 6D200-RTF1** | **5'-AGAACCTCTCGTCGCACTTC-3** | **RT-qPCR** | |
| **Ta 6D200-RTR1** | **5'-CCTTAGCCTGCCAAGCTCTT-3** | **RT-qPCR** | |
| **TaActinRTF** | **5'-GGAATCCATGAGACCACCTAC-3'** | **RT-qPCR** | |
| **TaActinRTR** | **5'-GACCCAGACAACTCGCAAC-3'** | **RT-qPCR** | |
| **BSMV-CPF** | **5'-TGACTGCTAAGGGTGGAGGA-3'** | **RT-PCR** | |
| **BSMV-CPR** | **5'-CGGTTGAACATCACGAAGAGT-3'** | **RT-PCR** | |
| **TaMPK3F** | **5'-TACATGAGGCACCTGCCGCAGT-3'** | **RT-qPCR** | |
| **TaMPK3R** | **5'-GGTTCAACTCCAGGGCTTCGTTG-3'** | **RT-qPCR** | |
| **TaERF3F** | **5'-GCAATCGGGCAAAGCAAAC-3'** | **RT-qPCR** | |
| **TaERF3R** | **5'-CGACTCAGAACGAACCACGA-3'** | **RT-qPCR** | |
| **TaDefensinF** | **5'-ATGTCCGTGCCTTTTGCTA-3'** | **RT-qPCR** | |
| **TaDefensinR** | **5'-CCAAACTACCGAGTCCCCG-3'** | **RT-qPCR** | |
| **TaChitinase3-F** | **5'-CCCACCCTAACCTGAGCATC-3'** | **RT-qPCR** | |
| **TaChitinase3-R** | **5'-ACTGGTTGATCATGGCGGAG-3'** | **RT-qPCR** | |
| **TaChitinase4-F** | **5'-GAAGTCCCCCATGGCGATC-3'** | **RT-qPCR** | |
| **TaChitinase4-R** | **5'-GGTCCCGCAATAACCGTACT-3'** | **RT-qPCR** | |
| **TaPR1-F** | **5'-AAACAGCAGCAACCCAAGAA-3'** | **RT-qPCR** | |
| **TaPR1-R** | **5'-GGGTCCAGTAGCACCGATTTA-3'** | **RT-qPCR** | |
| **NbActinF1** | **5'-TCCACGAGACTACATACAAC-3'** | **RT-qPCR** | |
| **NbActinR1** | **5'-GCCACCACCTTAATCTTCA-3'** | **RT-qPCR** | |
| **NbWIPKF1** | **5'-ACAGTTGAGGAAGCATTAGA-3'** | **RT-qPCR** | |
| **NbWIPKR1** | **5'-ATTCAGGATTCAGCGACAA-3'** | **RT-qPCR** | |
| **NbdefensinF1** | **5’-CATTGCTTGTCATGGCTAC-3'** | **RT-qPCR** | |
| **NbdefensinR1** | **5’-CACAGTTGCTATCTCTTGAG-3'** | **RT-qPCR** | |
| **NbERF3F1** | **5’-TTAACCTTGCTCCACCTAC-3'** | **RT-qPCR** | |
| **NbERF3R1** | **5’-TCTCTGTCTCACTCTTCTCT-3'** | **RT-qPCR** | |
| **NbPR1F1** | **5’-CTGCTAAGGCTGTTGAGAT-3'** | **RT-qPCR** | |
| **NbPR1R1** | **5’-TCCTCCATTGTTACACTGAA-3'** | **RT-qPCR** | |
